# Long-term ecological surveillance of hard ticks (Acari: Ixodidae) and SFTSV in Dangjin, South Korea (2018–2024)

**DOI:** 10.1186/s13071-026-07338-9

**Published:** 2026-03-30

**Authors:** Hyeon Jun Shin, Jun Yang Jeong, Chan Eui Hong, Hyeok Lee, Kyoung Won Lee, Wook-Gyo Lee, Jie Eun Park, Dae Kwon Song, Cho-I. Moon, Hee Ju Hwang, Yong Seok Lee

**Affiliations:** 1 Department of Biology, College of Natural Sciences, Soonchunhyang University, Asan-Si, Chungcheongnam-Do 31538 Republic of Korea; 2 Korea Native Animal Resources Utilization Convergence Research Institute (KNAR), Soonchunhyang University, Asan-Si, Chungcheongnam-Do 31538 Republic of Korea; 3 Research Support Center for Bio-Bigdata Analysis and Utilization of Biological Resources, Soonchunhyang University, Asan-Si, Chungcheongnam-Do 31538 Republic of Korea; 4 Education Project for Artificial Intelligence Integration in Biopharmaceutical Molecule Exploration, Soonchunhyang University, Asan-Si, Chungcheongnam-Do 31538 Republic of Korea; 5 Division of Vector and Parasite Diseases, Korea Disease Control and Prevention Agency (KDCA), Cheongju-si, Chungcheongbuk-do, Republic of Korea

**Keywords:** Tick surveillance, Ixodidae, SFTS virus, Vector-borne diseases, Seasonal dynamics

## Abstract

**Background:**

Hard ticks (Ixodidae) are the primary vectors of severe fever with thrombocytopenia syndrome virus (SFTSV), a tick-borne pathogen of increasing public health concern in East Asia. Understanding local vector ecology requires long-term monitoring, particularly in regions where human cases occur but viral prevalence in questing ticks remains unclear. This study conducted multi-year ecological and molecular surveillance of hard ticks and SFTSV in Dangjin-si (City), Chungcheongnam-do (Province), a representative region in west-central ROK.

**Methods:**

From 2018 to 2024, ticks were collected monthly from April to November across four habitat types (grassland, mountain road, mixed forest, and cemetery) using standardized 24-h CO_2_-baited traps. Specimens were morphologically identified, and a stratified subset was selected for molecular screening after stratification by habitat, species, developmental stage, and sex (for adults). Pooled samples were constructed within each stratum, with up to 50 larvae, 30 nymphs, or 5 sex-separated adults per pool. In total, 36,478 ticks were assembled into 3106 pools and screened for SFTSV by nested reverse transcription (RT)-PCR. Amplification products were evaluated by agarose gel electrophoresis.

**Results:**

Across the surveillance period, 72,956 ticks (adults, nymphs, and larvae) were collected. Among the collected ticks, three species were identified: *Haemaphysalis longicornis* (46,269), *H. flava* (1,143), and *Ixodes nipponensis* (655). *H. longicornis* was the most frequently collected and accounted for 63.42% of all adult and nymphal ticks. Tick abundance peaked during 2018–2019 and was highest in grassland habitats. SFTSV was not detected in any of the 3106 pools.

**Conclusions:**

Although SFTSV was not detected in the screened stratified subset, the persistently high abundance and broad ecological distribution of *Haemaphysalis* species indicate that Dangjin-si (City) maintains environmental conditions that may support pathogen introduction or amplification. These long-term data provide an ecological baseline for early warning and support targeted surveillance in nearby areas with higher incidence. Integrating ecological, climatic, and epidemiological data, together with multi-pathogen molecular screening, will strengthen One Health-based risk assessment in low-prevalence settings.

**Graphical Abstract:**

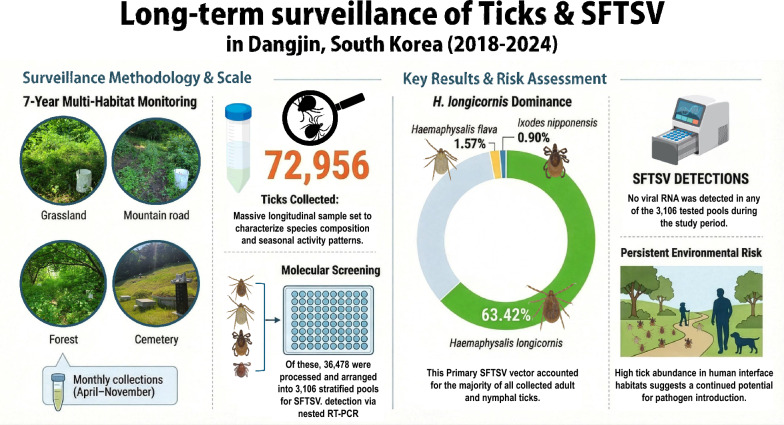

**Supplementary Information:**

The online version contains supplementary material available at 10.1186/s13071-026-07338-9.

## Background

Severe fever with thrombocytopenia syndrome (SFTS) is a tick-borne viral disease caused by *Dabie bandavirus* (family Phenuiviridae, genus *Bandavirus*) [1]. In the Republic of Korea (ROK), the primary vectors belong to the family Ixodidae, particularly *Haemaphysalis longicornis* and *H. flava*, which are widely distributed and frequently collected across diverse habitat types [2, 3]. Although tick bites and tick-borne diseases have long been recognized for decades, the association between *Haemaphysalis* ticks and severe viral infection was not established until the first confirmed human SFTSV case in 2012, with nationwide reporting beginning in 2013 [4–6]. Since then, SFTS has remained a significant public-health concern owing to its severe clinical manifestations, including high fever, thrombocytopenia, leukopenia, multi-organ dysfunction, and high mortality, especially among older populations [5, 7].

Seasonal variability is a major ecological driver influencing the distribution and activity of hard ticks. Recent predictive models suggest that the geographic expansion and phenology of *Haemaphysalis* species may shift under various seasonal scenarios [8–13]. In particular, *H. longicornis*, the dominant species in Korea, reaches high densities in grasslands, mountain roads, mixed forests, and cemetery landscapes, microhabitats that commonly overlap with human activity [13–15]. However, the direct pathway through which environmental change translates into increased human infection risk remains uncertain because tick–host–environment interactions depend on multiple factors, including wildlife host density, land-use patterns, vegetation structure, and human behavioral exposure.

Despite advances in SFTS epidemiology, long-term ecological observations of tick vectors in the ROK remain limited. Many previous tick surveys have been short-term, geographically restricted, or focused primarily on quantifying relative tick abundance rather than examining habitat-specific or interannual variation. Moreover, national surveillance has placed a strong emphasis on viral detection, whereas the ecological context, especially in regions with low human incidence of SFTSV, has been comparatively underexplored. This gap hampers the development of accurate risk assessments and early warning frameworks, particularly in areas where pathogen circulation may be sporadic or focal.

Dangjin-si (City), Chungcheongnam-do (Province), is a coastal agricultural area with extensive grasslands, mixed farmlands, cemetery complexes, and forest margins in the west-central part of the ROK and provides ecologically suitable environments for hard ticks, including *Haemaphysalis* and *Ixodes* species. Although the annual incidence of human SFTSV infections in this region is relatively low, the presence of suitable habitats and frequent wildlife–tick–human interface suggests that the area remains relevant for baseline ecological surveillance. Long-term non-detection of SFTSV in such ecologically favorable landscapes can still yield epidemiologically meaningful information by defining upper bounds of local transmission potential and establishing baseline thresholds applicable to low-prevalence settings.

Given these knowledge gaps, there is increasing recognition of the need for multidisciplinary and One-Health-aligned surveillance frameworks integrating vector ecology, environmental variables, and human incidence patterns [16–18]. In this context, the present study conducted a 7-year ecological monitoring program in Dangjin-si (City) (2018–2024) to (1) characterize long-term trends in tick abundance, species composition, and habitat-specific distribution; (2) examine seasonal activity patterns of dominant vector species; and (3) assess evidence for or against SFTSV circulation using extensive pooled molecular screening. By integrating vector dynamics with regional human incidence data, this study provides foundational ecological information essential for evaluating local transmission potential and strengthening future One-Health-based public health strategies.

## Methods

### Collection period, location, and environmental characterization

**Fig. 1:**
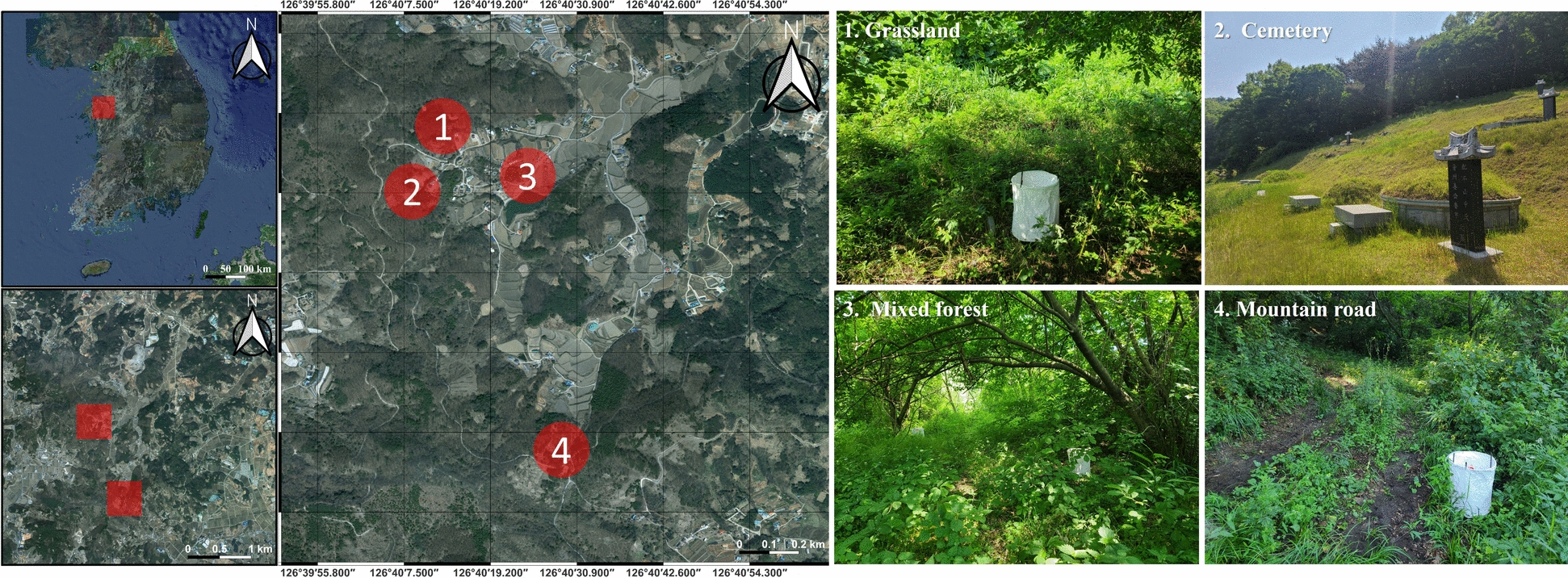
Surveillance areas and environmental types for hard tick collection

From 2018 to 2024, monthly tick surveillance was conducted from April through November, excluding December through March, across four habitat types (grassland, mountain road, mixed forest, and cemetery) in Dangjin-si (City), Chungcheongnam-do (Province), ROK (Fig. 1).

The surveillance period was selected to coincide with the seasonal activity of hard ticks, as *Haemaphysalis longicornis* typically enters diapause during late autumn and winter, whereas overall questing activity is markedly reduced during colder months. Although some species such as *Ixodes nipponensis* may remain active later in the year, winter sampling was not included in the standardized surveillance design that followed national monitoring procedures previously adopted in Korea [2, 14, 15]. The environmental characteristics of each site were documented, and GPS coordinates were recorded at the centroid of each habitat to ensure spatial reproducibility (grassland: 36°50′40.29″N, 126°40′11.64″E; mountain road: 36°50′02.20″N, 126°40′29.68″E; mixed forest: 36°50′37.72″N, 126°40′23.51″E; cemetery: 36°50′36.37″N, 126°40′09.55″E). The grassland, mixed forest, and cemetery sites were located adjacent to each other, whereas the mountain road site was relatively distant. All four habitats exhibited evidence of human residence or activity in the surrounding areas. The close spatial overlap between human land use and tick habitats within a limited area was a major criterion for selecting this region. The study area also supports diverse terrain features and hosts a variety of potential mammal reservoir species, including rodents (Muridae), deer (Cervidae), and canids (Canidae), which exhibit high mobility and habitat overlap. These characteristics make the site suitable for evaluating host–vector interactions and the local ecology of hard tick distributions [19–21]. The grassland habitat consisted of a flat area dominated by perennial grasses and herbaceous vegetation (approximately 30–100 cm in height), interspersed with occasional shrubs. The presence of water deer (*Hydropotes inermis*) was confirmed during field observations, indicating active vertebrate hosts. The mountain road consisted of a narrow, shaded path with a mixture of trees, shrubs, grasses, and herbaceous vegetation, showing clear signs of frequent human passage. The mixed forest contained decayed logs, leaf litter, and dense understory vegetation, forming a humid microhabitat suitable for a wide range of arthropods and small and large vertebrates. The cemetery consisted of open grassy areas with scattered gravestones, regularly cut grasses, and sparse tall vegetation bordered by adjacent forest habitat. Such environments are frequently visited by wildlife hosts, including water deer (*Hydropotes inermis*), likely owing to the presence of maintained grass cover, which may increase contact among wildlife hosts, ticks, and human activity. Seasonal human visitation, particularly during Chuseok when families tend gravesites, may coincide with periods of high larval abundance and thus represent a potential context of increased exposure.

Trap locations for each habitat are presented in Fig. 1 and were placed to avoid steep slopes and waterlogged areas. Within each habitat, three traps were positioned within a radius of approximately 40–50 m, maintaining a minimum inter-trap distance of 10 m. Whenever possible, identical trap locations were revisited across years (Fig. 1). Prior to trap placement, surrounding vegetation was cleared to ensure that the trap body was in direct contact with the ground.

**Fig. 2:**
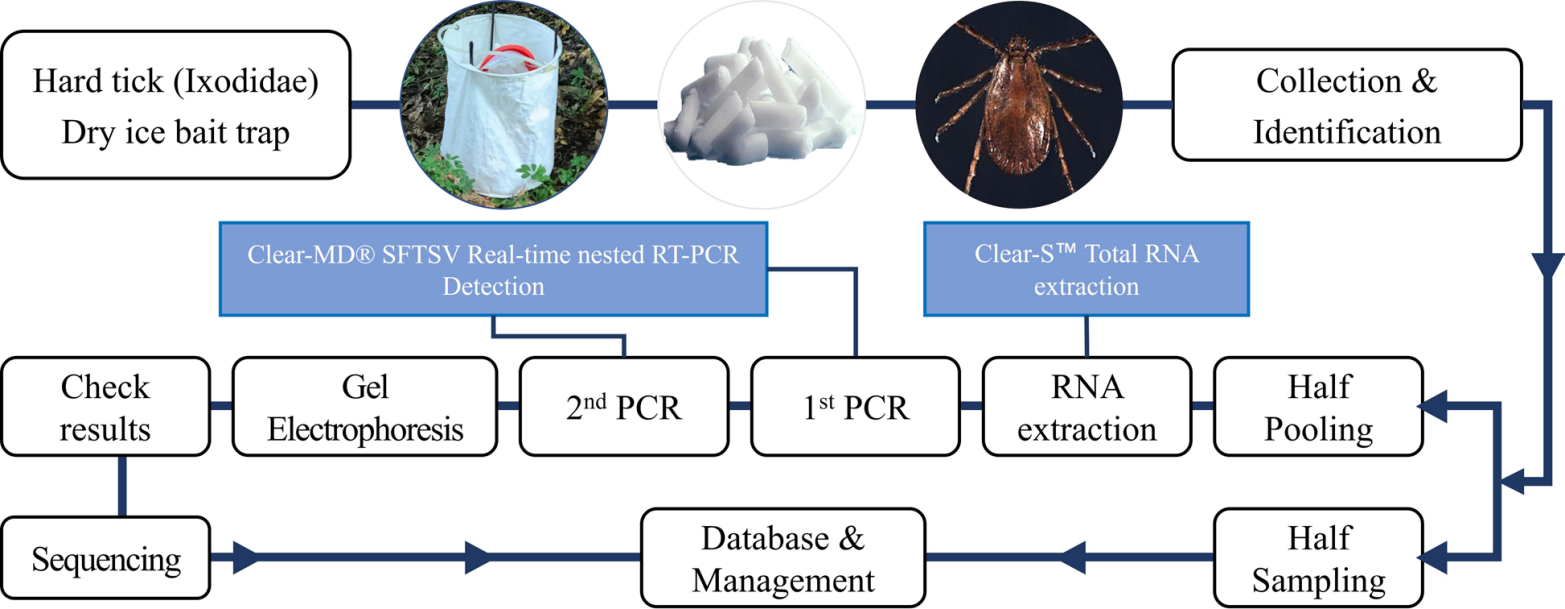
Workflow for hard tick surveillance, species identification, and molecular detection of SFTSV

Dry ice-baited traps consisted of insulated plastic beverage dispensers used as the trap body (Roichen MINI-B, 7 L; Roichen Co., Ltd., Republic of Korea) and were fitted with a commercially available white outer trap cover for hard tick collection (E-TND Co., Ltd., Republic of Korea) (Fig. 2). Each dispenser was filled with 3 kg of dry ice to provide a continuous source of sublimating CO_2_, which served as an attractant. The spout remained open to regulate CO_2_ emission, and all traps were deployed for 24 h. CO_2_-baited dry ice traps are recognized as an effective method for attracting hard ticks [22, 23].

After retrieval, each trap was sealed in a plastic bag and transported to the laboratory. Ticks and potential tick specimens attached to the interior and exterior of each trap were collected using a mechanical aspirator and fine forceps and placed into individual tubes. Each tick was handled individually; specimens were not pooled at the trap level, and debris from each trap was processed separately prior to individual specimen isolation. Collected specimens and associated debris from each habitat were then placed in a Petri dish over ice to minimize movement and examined under a stereomicroscope (Olympus SZ61, Tokyo, Japan). Non-tick arthropods, soil debris, plant fragments, and other particulates were removed, and only ixodid ticks were retained for further analysis.

### Species identification and developmental stage classification

Species identification and developmental stage classification were conducted using stereomicroscopes provided by the Research Support Center for Bio-Bigdata Analysis and Utilization of Biological Resources.

All ticks collected in the Republic of Korea (ROK) were identified to genus and species using the checklist of South Korean ticks and relevant taxonomic resources, including a standard morphological key and species descriptions for ticks recorded from South Korea [24–26]. Identification first separated specimens to family level as Argasidae or Ixodidae. Genera were then assigned primarily using the morphology of the anal groove and the capitulum. Species-level identification was subsequently completed using diagnostic external characters, including the mouthparts and leg segments, together with other relevant morphological traits [25, 26]. The identification workflow and decision criteria were reviewed and refined through annual internal training and proficiency assessments conducted in collaboration with the Korea Disease Control and Prevention Agency (KDCA). This training incorporated annual updates reflecting results from 16 regional sentinel surveillance sites, including year-to-year differences in vector occurrence and pathogen detection, and the current status of medically important vectors in Korea, whether imported or domestically occurring. Identified specimens were categorized by developmental stage (adult, nymph, larva). Adult ticks were additionally classified by sex.

Larvae of *Haemaphysalis longicornis* and *H. flava* exhibit highly similar morphological features that cannot be reliably differentiated under standard stereomicroscopic observation. Therefore, larvae with indistinguishable gnathosomal traits were grouped as *Haemaphysalis* spp. (larvae) to minimize errors in species-level distribution estimates for larval stages [27].

All specimens were cataloged by habitat, developmental stage, and sex. Half of the specimens from each group were used for RNA extraction and nested reverse transcription (RT)-PCR for pathogen detection, while the remaining half were preserved in 99% ethanol (EtOH) for verification of species occurrence and reproducibility checks. A complete overview of the workflow from field collection to pathogen analysis is shown in Fig. 2.

RNA extraction was performed for pooled tick samples prepared after each monthly field collection. All ticks collected in a given month were first separated by habitat. Within each habitat group, individuals were classified by species and developmental stage, and adult ticks were further separated by sex. Pools were assembled only after this stratification so that each pool represented a single habitat and a single biological category. All pools generated from all tick species and developmental stages, including *Haemaphysalis longicornis*, *H. flava*, *Ixodes nipponensis*, and morphologically unresolved *Haemaphysalis* larvae, were subjected to SFTSV screening.

This process was repeated independently each month throughout the 7-year study period, resulting in 3106 pools derived from a total of 36,478 ticks. Monthly stratified pooling was employed to minimize unnecessary dilution of viral RNA and to avoid mixing across ecologically distinct categories.

Pooling thresholds followed limits recommended by the manufacturer to ensure efficient homogenization and stable RNA extraction performance. Up to 50 larvae, 30 nymphs, or 5 adults (sex-separated) of a single species were placed into each extraction tube [28]. These limits reflected the maximum tissue load that could be fully lysed during homogenization while maintaining consistent extraction efficiency and adequate detection sensitivity.

Tick homogenization was conducted using the Clear-s Total RNA Extraction Kit (Invirustech, Republic of Korea, cat. no. IVT3001KS). β-Mercaptoethanol was added to the lysis buffer according to the manufacturer’s protocol. Samples were homogenized with a Precellys 24 instrument equipped with 2.8 mm zirconium beads at 7500 rpm for 30 s, followed by a 30 s pause, with the cycle repeated twice [29]. Subsequent RNA extraction steps were performed according to the manufacturer’s instructions. RNA was eluted in a final volume of 50 µL to maintain comparable concentrations across pools. Extracted RNA was kept on ice during processing and stored at −80 °C when not immediately used.

### PCR for SFTSV detection

SFTSV detection was performed using the Clear-MD^®^ SFTSV Real-time Nested RT-PCR Detection Kit (Invirustech, Republic of Korea, cat. no. IVT-M1002). All work surfaces, pipettes, and consumables were disinfected with 10% diluted bleach followed by 70% ethanol before and after each step to prevent contamination. Reaction mixtures were prepared according to the manufacturer’s protocol, and all reagents used in both the primary and secondary PCR reactions were supplied with the Clear-MD^®^ kit. Each reaction had a total volume of 20 µL and included the enzyme mix, SFTSV detection reagents, 10 µL of RNA template, and nuclease-free water as specified in the kit protocol [30, 31].

The assay consisted of two amplification rounds. Primary RT-PCR was performed using a Bio-Rad C1000 Touch™ Thermal Cycler (Bio-Rad, Hercules, USA) with reverse transcription at 50 °C for 15 min, enzyme activation at 95 °C for 3 min, followed by 40 cycles at 95 °C for 20 s, 60 °C for 20 s, and final extension at 72 °C for 40 s. The secondary nested PCR included enzyme activation at 95 °C for 3 min followed by 27 cycles at 95 °C for 15 s, 58 °C for 20 s, and 72 °C for 30 s. A positive control (kit-provided template) and a negative control (nuclease-free water) were included in each run.

The primer sequences used in this assay are proprietary and are not disclosed by the manufacturer. According to the kit documentation, the assay targets a single genomic segment of SFTSV; primers for other genomic segments (S, M, or L) are not included. The kit also contains proprietary primers intended for confirmatory sequencing, which were not used for independent segment-specific validation in this study.

PCR products were analyzed on a 1.5% agarose gel using a Bioneer AGARO power™ system with 1× TAE buffer. A mixture of 6 µL PCR product and 1 µL loading dye (Dyne LoadingSTAR+ , DyneBio, Republic of Korea) was loaded alongside a 100 bp DNA ladder. Electrophoresis was performed at 125 V (approximately 7 V/cm) for 50 min. According to the manufacturer’s interpretation criteria, the kit positive control produces an expected 219 bp band, whereas field samples are interpreted as positive when an amplicon of approximately 530 bp is observed. A valid run was defined by the presence of the expected 219 bp band in the positive control and the absence of amplification in the negative control. This difference in amplicon size reflects the assay design specified by the manufacturer and is not interpreted as misamplification. Analytical sensitivity was not determined experimentally in this study and is reported on the basis of manufacturer-provided validation materials. According to these data, the assay has a LoD95% of 1.643 viral copies per µL, with detection probabilities of approximately 99% at concentrations ≥ 2 viral copies per µL. Manufacturer-reported serial dilution experiments quantified by digital PCR (LOAA^®^ Dr.PCR system) indicate reliable detection across a broad dynamic range of viral RNA concentrations.

### Human SFTS case data

Human SFTS case data for Chungcheongnam-do (Province) (2015–2024) were obtained as aggregated administrative records from the Chungcheongnam-do (Province) Infectious Disease Control and Management Center (CNCIDC). Cases were summarized at the city/county level by year, and incidence rates were expressed per 100,000 population using the corresponding population denominators provided in the dataset. Quarterly distributions were classified as Q1 (January–March), Q2 (April–June), Q3 (July–September), and Q4 (October–December).

## Results

### Annual and habitat-specific tick collection numbers and proportions

From 2018 to 2024, a total of 72,956 hard ticks were collected across the four surveyed habitats (grassland, mountain road, mixed forest, and cemetery) in Dangjin-si (City), Chungcheongnam-do (Province). Annual collection numbers were highest in 2018 (*n* = 16,996) and 2019 (*n* = 21,668) and showed a gradual decline thereafter: 9086 in 2020, 8769 in 2021, 5168 in 2022, 5953 in 2023, and 5316 in 2024.

**Fig. 3:**
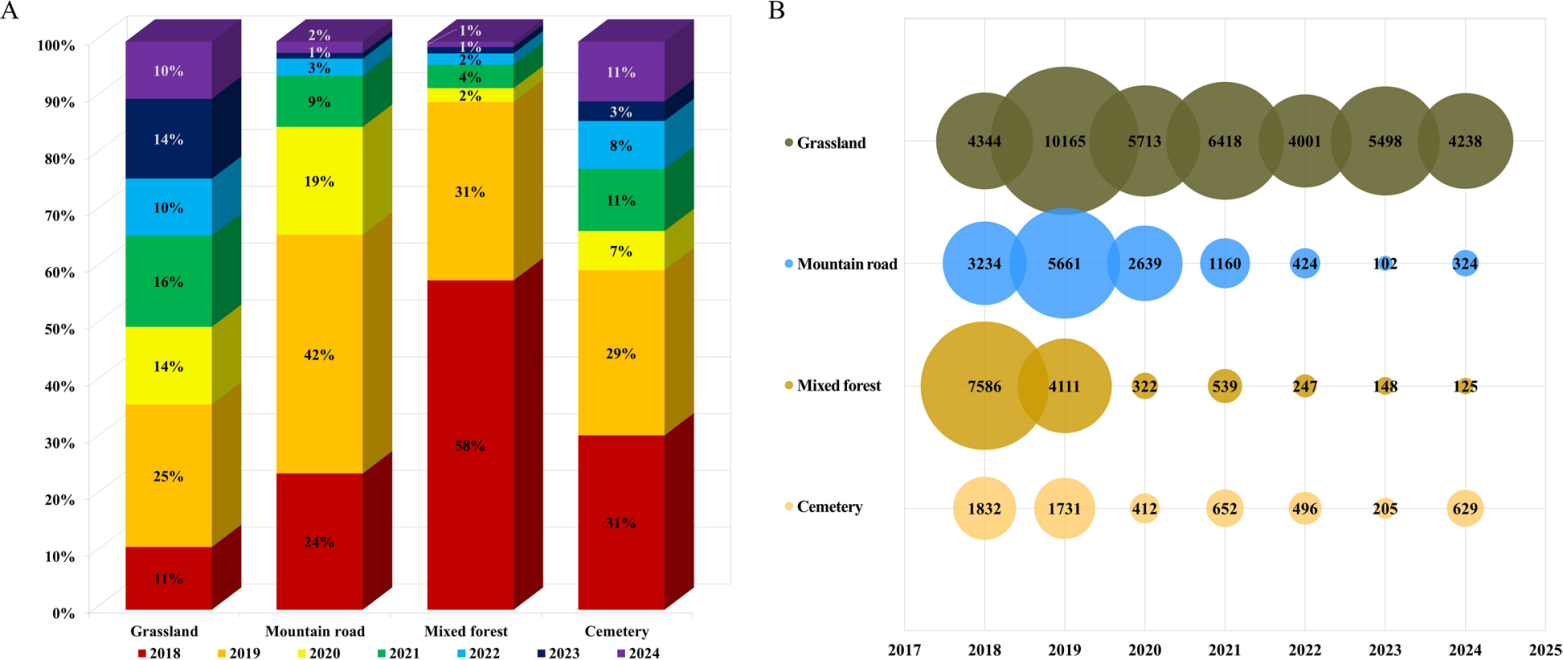
Annual and habitat-specific abundance and composition of hard ticks collected (2018–2024). (**A**) Annual totals of hard ticks collected in each habitat (grassland, mountain road, mixed forest, and cemetery). Values indicate the total number of collected ticks across all developmental stages. (**B**) Annual proportional composition of hard ticks by habitat, expressed as percentages of the annual total. Monthly occurrence by species (April–November)

**Fig. 4:**
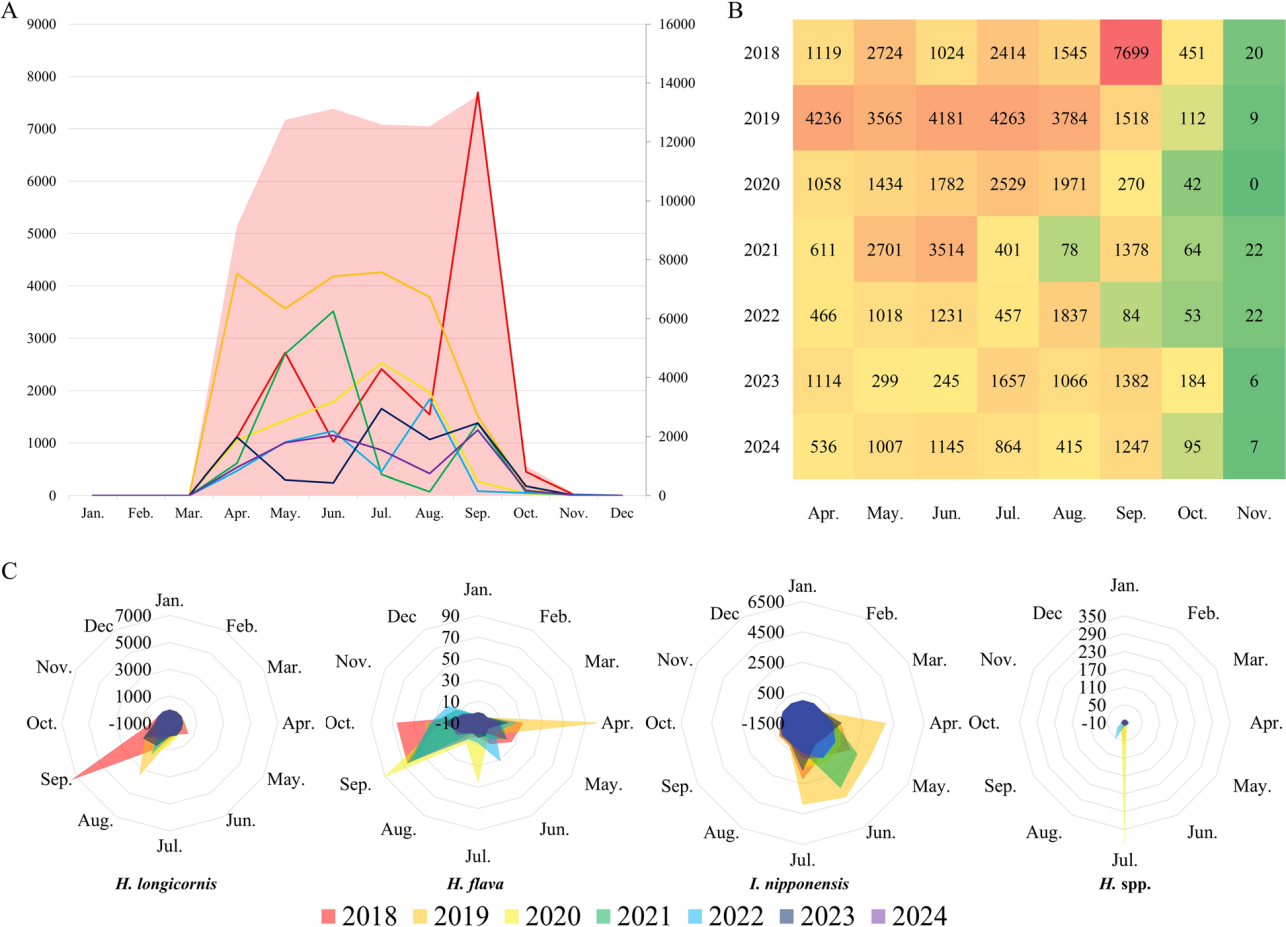
Seasonal and interannual patterns of monthly tick collections by taxon and life stage (2018–2024). Summarizes monthly tick collections from April to November across years, including total counts (**A**), a heatmap of monthly totals (**B**), and species- or stage-specific monthly patterns (**C**)

Habitat-specific patterns indicated that the grassland habitat yielded the highest number of ticks, accounting for 42,186 ticks (56.40%), followed by the mountain road (13,555; 18.12%), mixed forest (13,092; 17.50%), and cemetery (5965; 7.97%) (Fig. 3A, B). A notable surge in tick abundance was observed in the grassland and mountain road habitats in 2019, after which a marked decline occurred across most habitats (Figs. 3A, B and 4A, B).

Monthly and species-specific analyses of ticks collected between 2018 and 2024 showed clear seasonal and interannual variation (Fig. 4A–C). *Haemaphysalis longicornis* was the most frequently collected species and peaked between April and July, with notable increases in 2019 and 2021. *Haemaphysalis flava* occurred at lower frequency and was recorded from April to October, with higher counts in September and October. *Ixodes nipponensis* was consistently infrequent throughout the study period, with low monthly counts across years. Larval *Haemaphysalis* spp. (larvae not reliably distinguishable between *H. longicornis* and *H. flava*) increased in late summer to early autumn, including pronounced peaks in September 2018 and September 2019 (Fig. 4C).

Of all ticks collected (*n* = 72,956), *H. longicornis* (adults and nymphs) accounted for 46,269 (63.42%), followed by *Haemaphysalis* spp. (larvae, 24,889, 34.12%), *H. flava* (1143, 1.57%), and *I. nipponensis* (655, 0.90%).

## Discussion

### Ecological interpretation of long-term tick surveillance data

This study provides a 7-year ecological dataset characterizing the distribution, seasonal activity, and habitat-specific occurrence of hard ticks in Dangjin-si (City), Chungcheongnam-do (Province). Three tick species were identified, with *Haemaphysalis longicornis* representing the majority of adults and nymphs collected. This dominance is consistent with nationwide surveillance findings reporting *Haemaphysalis* species as the prevailing ticks in rural and peri-urban landscapes across the ROK [14, 15]. The persistently high proportion of *H. longicornis* across all four habitat types suggests broad ecological tolerance and effective utilization of a diverse vertebrate host community. However, previous studies have shown that *H. longicornis* is primarily associated with medium-to-large-sized mammals, whereas rodents more frequently host species such as *Ixodes nipponensis*. This host association suggests that rodents may contribute less to the maintenance of *H. longicornis*-associated transmission cycles in the ROK.

Seasonal variation in activity patterns observed in this study is consistent with the established life cycle of *H. longicornis*. Nymphs predominated during spring, followed by increased adult activity in early to mid-summer and a pronounced larval peak in autumn prior to diapause. The monthly distribution of total tick collections (Fig. 4A) showed that overall abundance was concentrated between April and October, with a rapid decline after September and minimal collections during winter months. Peak timing and magnitude varied among years, including a marked increase in September 2018. *H. flava* exhibited relatively later seasonal activity peaks in September and October, while larval *Haemaphysalis* spp. were most abundant during August and September. These seasonal windows overlap with periods of increased agricultural, forestry, and outdoor human activity, thereby increasing opportunities for human–tick encounters [3]. Importantly, transovarial transmission of SFTSV in *H. longicornis* has been documented previously, underscoring the epidemiological relevance of autumnal larval peaks even in the absence of detectable infection in questing adults [32, 33].

The temporal heatmap (Fig. 4B) further indicates that agricultural fields, forest margins, and peri-urban interfaces may sustain extended periods of exposure risk throughout the warm season owing to overlapping activity windows of multiple tick life stages. Similar patterns have been reported from other regions in East Asia, where the coexistence of larvae, nymphs, and adults extends the seasonal window of potential pathogen transmission [34, 35].

Interannual variation also revealed meaningful ecological signals. Total tick abundance peaked during 2018–2019 and declined gradually thereafter (Figs. 3 and 4). These fluctuations may reflect a combination of factors, including repeated sampling at fixed sites changes in vegetation structure, land-cover transitions, and variation in wildlife host availability. In addition, sustained human activity at collection sites may have influenced the local presence or movement of medium-sized mammals, such as water deer, indirectly affecting tick population dynamics. Because such interannual processes cannot be resolved through short-term surveys, these results highlight the value of long-term surveillance for accurately interpreting local vector ecology.

The habitat distribution shown in Figs. 3 and 4 illustrates that landscapes characterized by forest margins, agricultural fields, and residential zones function as interface environments where ticks, wildlife hosts, domestic animals, and humans interact frequently. Dangjin-si (City) contains a mosaic of such environments that supports abundant tick populations closely associated with human activities. These observations reinforce the relevance of a One Health framework that integrates environmental, veterinary, and human health components when assessing future tick-borne disease risks.

### Interpretation of negative SFTSV detection and factors influencing viral detectability

Although 36,478 ticks grouped into 3106 pools were tested using nested RT-PCR, SFTSV was not detected in any of the pools. This finding is consistent with several surveillance studies conducted in the ROK, which reported extremely low or undetectable SFTSV prevalence in questing ticks even in areas with high vector abundance [36–38]. Given the large number of pools analyzed and the inherent dilution effect associated with pooled testing, these results are most reasonably interpreted as reflecting either very low-level viral circulation or viral activity below the operational detection threshold of pooled surveillance.

The analytical sensitivity of the assay further supports this interpretation. According to the manufacturer, the nested RT-PCR system has a detection limit of LoD 95% = 1.643 viral copies per microliter and achieves approximately 99% detection probability at concentrations of two or more copies per microliter. Given this level of sensitivity, the absence of positive detections suggests that the presence of SFTSV in questing ticks was likely distributed at concentrations below detectable levels when diluted across pooled samples. Thus, the negative results should be interpreted as reflecting methodological and ecological constraints rather than evidence of true viral absence.

Broader comparative patterns across East Asia reinforce this view. While China and Japan frequently report SFTSV-positive ticks across diverse ecological settings, Taiwan consistently reports no viral detection in ticks despite ongoing human SFTSV surveillance [39–41]. These contrasting regional patterns indicate that SFTSV circulation is shaped by fine-scale ecological conditions and host-associated transmission networks rather than by vector density alone. The micro-focal and intermittent nature of SFTSV transmission increases the likelihood that environmental sampling may miss viral hotspots that are spatially restricted or temporally transient.

Importantly, negative molecular detection does not equate to confirmed viral absence. Non-detection may occur when infection prevalence is extremely low, when viral circulation is confined to micro-focal hotspots outside designated sampling sites, or when RNA degradation occurs prior to laboratory processing. Pooling further reduces the probability of detecting SFTSV infected individuals by diluting viral RNA below detectable concentrations [42, 43]. Such limitations are well recognized in tick-borne pathogen surveillance, where transmission is often shaped by wildlife host movements and microclimatic variability operating at spatial scales smaller than those captured by standard environmental sampling frameworks [27, 44, 45].

Because the nested RT-PCR assay used in this study targets a single genomic segment of SFTSV, the possibility of segment-specific discordance cannot be excluded. Previous field studies have reported partial detection of individual SFTSV genome segments in ticks, indicating that infection may not always be uniformly detectable across all segments. Accordingly, the absence of detectable SFTSV RNA in this study should be interpreted in the context of assay design and does not preclude the presence of low-level or segment-limited viral circulation in the environment.

The divergence between environmental tick surveillance and human case occurrence in Dangjin-si (City) illustrates these constraints. Although no SFTSV was detected in ticks collected from this area, approximately 12 human SFTS cases were reported during the study period. A similar discrepancy has been reported in Oita Prefecture, Japan, where one region showed no evidence of SFTSV or antiviral antibodies in ticks, wildlife, livestock, or humans, despite clear viral activity in surrounding areas [46]. These observations provide further evidence that tick-based environmental surveillance alone may underestimate transmission risk when viral circulation is highly localized or maintained within specific host–vector networks.

**Fig. 5:**
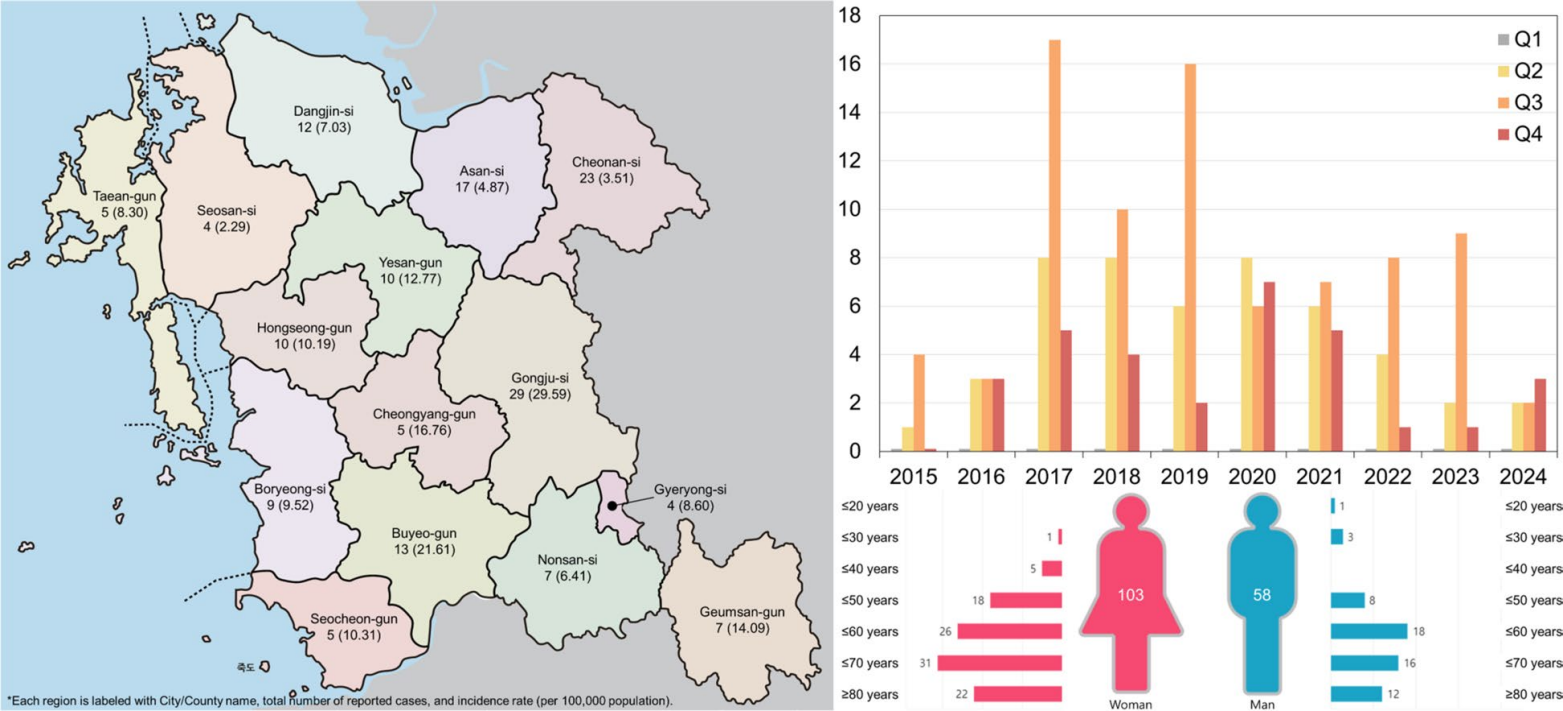
Spatial, temporal, and demographic patterns of human SFTSV cases in Chungcheongnam-do (Province), Republic of Korea (2015–2024). Data were obtained from the Chungcheongnam-do (Province) Infectious Disease Control and Management Center (CNCIDC). Each administrative region is labeled with the city or county name, total number of reported cases, and incidence rate per 100,000 population. Quarterly distributions are shown as Q1 (January–March), Q2 (April–June), Q3 (July–September), and Q4 (October–December)

Consistent with this interpretation, human SFTS incidence in Chungcheongnam-do (Province) displays pronounced spatial heterogeneity (Fig. 5). Regional comparisons based on incidence rates rather than absolute case counts reveal that Gongju-si (City), Cheonan-si (City), and Asan-si (City) exhibit substantially higher incidence rates than Dangjin-si (City), suggesting uneven distribution of ecological and epidemiological conditions that support viral maintenance. Seasonal patterns further indicate concentration of cases in specific quarters of the year. Together, these findings suggest that aligning future tick collection efforts with spatial patterns of human incidence and seasonal peaks may improve the strategic targeting of surveillance and the precision of One-Health-oriented risk assessments [47]. Use of multi-segment molecular assays may therefore improve detection sensitivity in future surveillance, particularly in settings where viral prevalence is expected to be extremely low. Such methodological refinements, combined with integrated ecological and epidemiological data, may contribute to a more accurate understanding of SFTSV transmission dynamics and support the development of more effective early warning and surveillance strategies.

### Implications for public health and future surveillance directions

The ecological patterns identified in this study carry important implications for public health planning and the refinement of national tick-borne disease surveillance strategies. Although no SFTSV was detected in questing ticks from Dangjin-si (City), the consistently high abundance of *Haemaphysalis* species across all habitat types indicates that ecological conditions conducive to viral introduction or amplification remain present. Similar situations have been reported in other regions where vector densities are high but viral circulation is absent or intermittent, suggesting that transmission risks are influenced not only by tick abundance but also by host community structure and fine-scale environmental variability [14, 16, 36].

Recent studies further indicate that integrating ecological surveillance with complementary environmental and spatial datasets can enhance identification of micro-focal risk areas for vector-borne diseases. Although such approaches were not applied in the present study, they may be valuable for future SFTSV surveillance in Korea, particularly in heterogeneous landscapes such as Dangjin-si (City), where agricultural, forested, and peri-urban habitats intersect. Beyond the species detected in this study, consideration of vectors not observed locally is also important for interpreting regional transmission risk. *Amblyomma testudinarium*, a recognized SFTSV vector in southern Korea, was not detected during the present study; however, recent reports from central regions, including areas near Daejeon, indicate that this species has been increasingly recorded beyond its historically recognized range [48]. While the drivers of this pattern remain uncertain, continued surveillance across broader latitudinal gradients will be necessary to determine whether these records reflect sustained distributional shifts or localized introductions with limited epidemiological relevance.

This study has several limitations that should be considered when interpreting the results. Surveillance was conducted within a single city and relied on CO_2_-baited traps at fixed sites, which may not fully capture habitat-specific or host-associated tick populations. Repeated removal sampling at the same locations may have contributed to interannual declines in tick abundance independent of broader ecological change. Molecular screening was performed using pooled samples and a single commercial nested RT-PCR target, which may reduce sensitivity for detecting low-level viral circulation and does not account for potential segment-specific discordance. In addition, human SFTS case data were available only at an aggregated administrative level, without individual exposure or activity histories, limiting spatial linkage between tick collection sites and transmission locations.

## Conclusions

This study provides a 7-year longitudinal assessment of hard tick ecology and One-Health-based pathogen risk in Dangjin-si (City), a representative region of west–central ROK. Although SFTSV was not detected in any of the 3106 pools, the persistent dominance of *Haemaphysalis longicornis* across years and habitat types indicates that local ecological conditions remain suitable for the maintenance or potential introduction of tick-borne pathogens. Accordingly, the absence of viral detection should not be interpreted as reduced transmission risk, particularly given the proximity of Dangjin-si (City) to neighboring regions with higher incidence.

The long-term dataset established here offers a valuable ecological baseline for evaluating future changes in tick species composition, population dynamics, and pathogen circulation. Strengthening surveillance through the integration of ecological context, complementary sampling strategies, and multi-pathogen molecular screening may enhance early-warning capacity for SFTSV and related tick-bone diseases within a One Health framework.

## Supplementary Information

Additional file 1.

## Data Availability

All data generated or analyzed in this study are included in this published article and its supplementary files.
